# AECHL-1 targets breast cancer progression via inhibition of metastasis, prevention of EMT and suppression of Cancer Stem Cell characteristics

**DOI:** 10.1038/srep38045

**Published:** 2016-12-15

**Authors:** Aparajita Dasgupta, Mithila A. Sawant, Gayatri Kavishwar, Manish Lavhale, Sandhya Sitasawad

**Affiliations:** 1 National Centre for Cell Science, NCCS Complex, S.P. Pune University, Ganeshkhind, Pune 411007, Maharashtra, India; 2 Pharmazz India Private Limited, H-6, Site-C, Surajpur Industrial area, Greater Noida, UP- 201307, India

## Abstract

Triple negative breast cancer (TNBC) features among the most aggressive manifestations of cancer due to its enhanced metastatic potential and immunity to therapeutics which target hormone receptors. Under such scenarios, anti-cancer compounds with an ability to influence multiple targets, or an entire process, will have an advantage over specific signal transduction inhibitors. To counter the metastatic threat it is essential to target cellular components central to the processes of cancer cell migration and adaptation. Our previous work on a novel triterpenoid, AECHL-1, explored its anti-cancer potential, and linked it to elevated ER stress in cancer cells, while its anti-angiogenic potential was credited for its ability to manipulate the cytoskeleton. Here, we broaden its range of action by showing that it curbs the metastatic ability of TNBC cells, both *in vitro* in MDA-MB-231 cell line and *in vivo*, in mouse models of metastasis. AECHL-1 does so by disrupting the cytoskeletal network, and also suppressing NF-κB and β-Catenin mediated key molecular pathways. These activities also contributed to AECHL-1 mediated suppression of TGF-β/TNF-α induced Epithelial to Mesenchymal Transition (EMT) and cancer stem cell characteristic. Thus, we present AECHL-1 as a promising therapeutic inhibitor of metastatic disease.

The tumor and its microenvironment are a hub of dynamic cellular activities. Several molecular processes are orchestrated in a cancer cell in response to peripheral stimuli, which lead to cancer establishment and progression. Irrespective of the advances in clinical and preclinical trials of cancer therapy, breast cancer remains one of the leading causes of mortality in women, with most of the fatalities being attributed to its metastasis^1^,^2^. Difficulties in the treatment of metastasis are attributed mainly to the heterogeneous nature of tumor cells and their interactions with the microenvironment.

To metastasize, the cancer cell remodels the cytoskeleton and forms membrane protrusions, at the leading edge, thus initiating invasion and migration^3^,^4^,^5^. The nexus of migration-invasion-metastasis is often associated with the process of epithelial to mesenchymal transition (EMT)^6^, which is characterized by the loss of epithelial markers, like E-cadherin and gain of mesenchymal markers, such as N-Cadherin, Vimentin, Snail and Twist^7^,^8^. Associated perpetrators of breast cancer relapse, the cancer stem cells (CSCs) harbor an enhanced ability to escape chemo/radio-therapy, and an up-regulation of CSC markers has been reported to be intimately linked to the process of EMT^9^,^10^. These interlinked mechanisms of EMT and metastasis have a cumulative effect in augmenting the complexity of the disease, and hence targeting them is of paramount importance.

Increase in the cases of relapse and resistance have elicited the need for the development of chemotherapeutics with strategic modes of action^11^. Natural compounds have been shown to possess enormous potential as anti-proliferative as well as anti-metastatic agents against multiple cancer types^12^,^13^,^14^. Earlier, we had reported the anticancer activity of a novel triterpenoid, AECHL-1, isolated from the root bark of *Ailanthus excelsa* RoxB^15^ and recently we gained further insights into its mechanism of action, and demonstrated that AECHL-1 could trigger apoptosis in breast cancer cells via mitochondrial perturbations and elevated ER stress^16^. Another line of investigation revealed that AECHL-1 inhibits tumor angiogenesis of breast cancer cells via cytoskeletal disruption^17^.

In the present study, we sought to determine the anti-migratory and anti-invasive potential of AECHL-1 on TNBC MDA-MB-231 cells and in mice models of tumorigenesis and metastasis. Our findings demonstrate that AECHL-1 could inhibit cancer cell migration and invasion by targeting the processes of actin nucleation and branch formation, both *in vitro* and *in vivo*. AECHL-1 could also suppress the phenomenon of EMT and reduce the expression of CSC indicators. AECHL-1 could execute these activities by down-regulating the expression of proteins such as β-catenin and NF-κB, which are engaged in regulation of the above mentioned processes, thus making AECHL-1 an effective dispenser of anti-cancer activities.

## Results

### AECHL-1 inhibits TNF-α mediated MDA-MB-231 migration and invasion through down-regulation of MMP-9 activity

**Figure 1:**
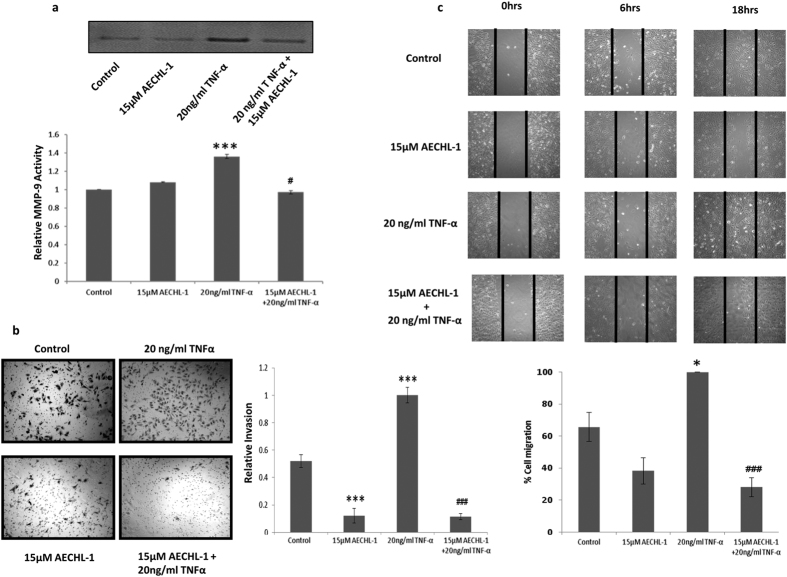
AECHL-1 inhibits migration, invasion and MMP-9 activity of breast cancer cells. (**a**) AECHL-1 decreased MMP-9 activity as detected by Gelatin zymography of cell supernatants. (**b**) AECHL-1 inhibited cancer cell invasion. Cells on the lower side of the membrane were considered. Cells were photographed (magnification, 10X) using Image pro plus and quantification was carried out using Image J software for all above described experiments. (**c**) AECHL-1 inhibited MDA-MB-231 migration. Confluent monolayer was scratched by pipette tip and treated with AECHL-1 in the presence or absence of 20ng/ml TNF-α. Migration is expressed as % gap closure of TNF-α treated well. Columns, mean from three independent experiments; bars, SE. *P < 0.05; ***P < 0.001; versus control and ^#^P < 0.05; ^###^P < 0.001 versus TNF-α.

In order to determine whether AECHL-1 affected invasiveness, MDA-MB-231 cell line was chosen, for its established invasive potential and mesenchymal phenotype. Matrix Metallo Proteinase (MMP)-9 activity, essential for the cells to digest the ECM in order to migrate or invade, was studied following TNF-α induction. 15 μM AECHL-1 treatment reduced MMP 9 activity as determined by zymography (Fig. 1a). This result also explained and strengthened our observation concerning AECHL-1 mediated inhibition of transwell invasion (Fig. 1b) and, migration across a scratch wound (Fig. 1c).

### AECHL-1 hampers breast cancer cell invasion through suppression of NF-κB mediated MAPK activity and decreases mesenchymal marker expression

**Figure 2:**
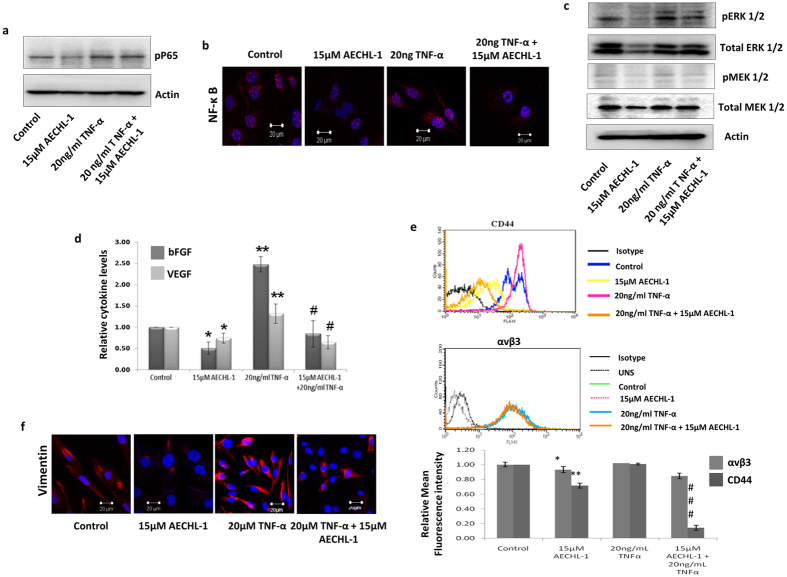
AECHL-1 down regulates TNF-α induced MAPK pathway downstream of NF-κB. MDA-MB-231 cells were grown in plates or coverslips and treated as indicated. AECHL-1 decreased pp65 nuclear expression and localization, as detected by western blotting (**a**) and IF (**b**) studies. (**c**) AECHL-1 also inhibited MAPK activation in the presence or absence of TNF-α induction. Images are representative of three independent experiments. The full length blots and densitometry analyses are given as supplementary Figure 1 and 2 respectively. AECHL-1 suppresses the expression and secretion of mesenchymal markers despite TNF-α stimulation. (**d**) ELISA based analysis demonstrated that AECHL-1 restricted the secretion of pro-angiogenic cytokines into MDA-MB-231. (**e**) Flow cytometry analysis of CD-44 and αvβ3 revealed an AECHL-1 effected decrease in their expression levels in MDA-MB-231 cells. (**f**) IF studies utilizing a confocal microscope (X 60) too showed a decrease in Vimentin expression. C.M. Images are representative of three independent experiments. Columns, mean from three independent experiments; bars, SE. *P < 0.05; **P < 0.01; versus control and ^#^P < 0.05; ^###^P < 0.001 versus TNF-α.

NF-κB is an important regulator of many pro-survival, invasive and inflammatory pathways^18^,^19^. The status of NF-κB was determined in these cells after 15 μM AECHL-1 treatment, in the presence or absence of 20 ng/ml TNFα. A downregulation in phosphorylated p65 subunit of NF-κB was seen by western blotting (Fig. 2a). Immunofluorescence studies revealed decrease in p65 localization in the nucleus (Fig. 2b). Whole cell protein lysates of treated MDA-MB-231 were also analyzed for the expression of pERK 1/2 and pMEK 1/2 via Western Blotting. This revealed a significant decrease in the expression and activity of MAPK proteins as phosphorylated forms of both the proteins were reduced by AECHL-1 treatment (Fig. 2c).

Since secretion of pro-angiogenic factors elicits a pro-metastatic response from tumor cells and may induce them to switch phenotypes from epithelial to mesenchymal, thus initiating migration and invasion^20^, cells were exposed to AECHL-1 treatment 2 h prior to TNF-α induction. Following termination, cells were analyzed for expression of invasion and mesenchymal markers through either flow cytometry or confocal microscopy. Pro-angiogenic/invasive growth factor secretion was studied by ELISA. AECHL-1 remarkably decreased bFGF/VEGF secretion into the conditioned media (CM) in the presence and absence of TNF-α induction as determined by ELISA (Fig. 2d).

Vimentin, CD-44 and αvβ3 are characteristic markers usually sported by the cancer cells having a mesenchymal phenotype^21^,^22^,^23^. Reduction in CD-44 and αvβ3 expression (Fig. 2e) was observed following TNF-α induction. AECHL-1 treatment also brought about a decrease in Vimentin expression, as observed by immunofluorescence analysis (Fig. 2f).

### AECHL-1 affects cancer cell migration by altering cytoskeletal dynamics

**Figure 3:**
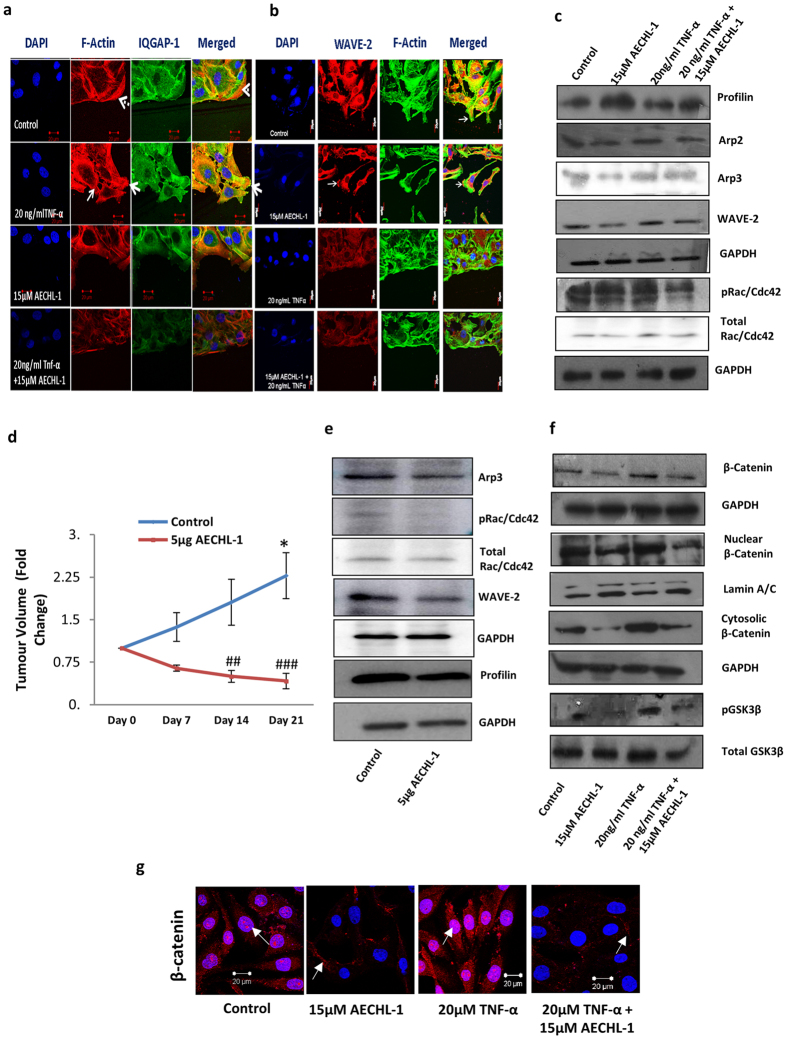
AECHL-1 affects cytoskeletal organization and assembly, *in vitro* and *in vivo*. Cells were grown till confluency either on coverslips or dishes and 9 h following a scratch wound, they were processed for immunofluorescence studies or western blotting. MDA-MB-231 stained with (**a**) phalloidin-Alexa Fluor647 (colored: red) and anti IQGAP1 antibody (**b**) phalloidin-Alexa Fluor488 and anti-WAVE-2 antibody were imaged using a confocal microscope (X 60). Membrane ruffles indicate active lamellopodial edge (white arrow). (**d**) Graph representing regression of MDA-MB-231 xenograft tumor volumes post AECHL-1 treatment. (**c**) Western blotting of cytoskeletal assembly related proteins *in vitro* and (**e**) *in vivo* (n = 5 mice per group). GAPDH was used as a loading control. AECHL-1 prevents β-catenin stabilization, *in vitro*. Cells were grown till confluency either on coverslips or dishes and 9 h following a scratch wound, they were processed for western blotting. (**f**) Western blotting for detection of β-catenin, GSK-3β, pGSK-3β in whole cell lysates and nuclear/cytoplasmic extracts. Blots were stripped and reprobed for GAPDH to indicate equal loading. (**g**) β-catenin nuclear (arrowhead) and cytoplasmic (white arrow) localization was captured using a confocal microscope (X 60). Images are representative of three independent experiments. The full length blots and densitometry analyses are given as supplementary Figure 1 and 2 respectively.

AECHL-1, in our previous study, had revealed an ability to de-regulate the actin cytoskeletal dynamics in endothelial cells^17^. An observed decrease in migration and invasion by MDA-MB-231 cells on AECHL-1 treatment prompted us to explore this phenomenon further. *In vitro* experiments involved a typical scratch wound assay where cells were initially exposed to AECHL-1 for 2 h following a scratch infliction and TNF-α induction. Experiments were terminated at 9 h following wounding. Cells were then lysed in RIPA and subjected to western blotting in order to study the expression of proteins involved in actin nucleation and branching during cancer cell migration. AECHL-1 could inhibit F-actin polymerization in migrating cells, and affected the localization of IQGAP-1 and WAVE-2 (Fig. 3a,b). AECHL-1 could also downregulate proteins belonging to the Rho family of small GTPases-Rac/cdc42, and the actin branch generators ARP-2/3 (Fig. 3c). Interestingly, profilin another important protein known to be instrumental for the rapid polymerization of the cytoskeleton^24^,^25^ was upregulated following AECHL-1 treatment.

Our *in vivo* results too displayed a similar trend. 5 μg/kg body weight AECHL-1, along with a significant regression in MDA-MB-231 xenograft tumor volume, downregulated the expression of actin nucleation and branching proteins with respect to PBS treated control (Fig. 3d,e). Profilin, however was found to be decreased in AECHL-1 treated mice, suggesting that profilin expression and translation may be situation dependent.

β-catenin accumulation in the nucleus is often associated with loss of E-cadherin and decrease in CD-44 expression. This correlates with susceptibility of the cell towards undergoing EMT, and acquisition of an invasive phenotype^26^. β-catenin dynamics at the membrane is also affected by Rac/Cdc42 GTPase activity involving alteration of IQGAP1 affinity with this protein. This phenomenon alters cell-cell adhesion and contacts, thus modifying cell polarity and shape. Since a change in morphology and cell-cell attachment was observed after AECHL-1 treatment, the status of β-catenin was also studied *in vitro*. β-catenin levels were downregulated after AECHL-1 treatment and nuclear localization was decreased (Fig. 3f). It was observed that there was a slight membrane localization of β-catenin following AECHL-1 treatment, indicating an attempt by the cells towards maintenance of junctional integrity (white arrow, Fig. 3g). GSK-3β is a modulator of β-catenin stability and is known to mark β-catenin for degradation by phosphorylating it. Phosphorylation of GSK-3β by Caesin kinase and AKT deactivates it^26^. As expected, AECHL-1 treatment could bring down phosphorylation of GSK-3β, thus preserving the effective GSK-3β levels, which would in turn mark β-catenin for ubiquitination, in consequence restricting its nuclear localization (Fig. 3g)

### AECHL-1 inhibits metastasis of MDA-MB-231 cells in an *in vivo* tail-vein mouse model

**Figure 4:**
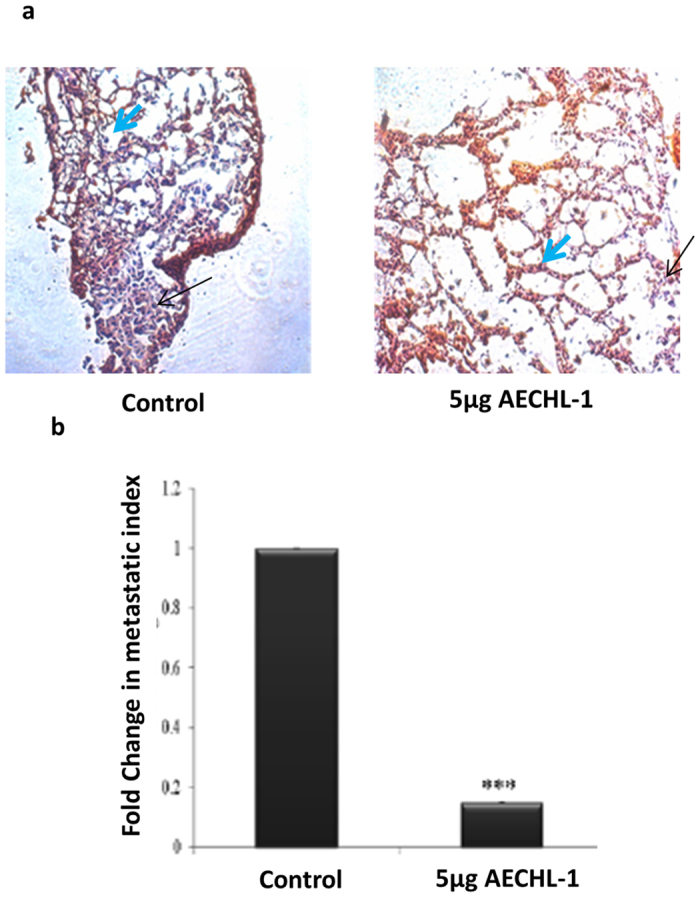
AECHL-1 inhibits generation of metastatic foci by MDA-MB-231 *in vivo*. Control/ PBS treated mice show presence of MDA-MB-23-Luc metastasis to the lungs. Weeks are numbered post AECHL-1 treatment completion. (**a**) H&E stained cryosection of lungs (n = 5 mice per group). Small blue arrow; normal lung morphology. Long black arrow; metastatic foci. Images were taken using a 20 X objective in bright field. (**b**) Fold change in metastatic index. Columns, mean from 5 mice per group; bars, SE. ***P < 0.001 versus control.

SCID female mice were inoculated with MDA-MB-231 cells via tail vein injection, and 5 μg/kg body weight of AECHL-1 was administered to the mice intra-peritoneal (i.p.) for the duration of 10 days. Control mice were treated with PBS. Lungs were excised after the duration of 4 weeks and studied for morphological characteristics typical of affected lungs. They were then processed for H&E staining to observe metastatic foci. Lungs from AECHL-1 treated mice showed normal alveolar appearance with sparse metastatic foci, whereas lungs excised from the PBS treated control group sported larger numbers of dense metastatic foci (Fig. 4a). We further quantified the metastatic focal density by grading them according to the number and continuity per sample. It was observed that AECHL-1 could decrease this parameter in the lungs of treated mice. Thus AECHL-1 could decrease metastatic colonization by MDA-MB-231 cells in the lungs of treated mice, as depicted by the images (Fig. 4b).

### AECHL-1 discourages Epithelial to Mesenchymal Transition

**Figure 5:**
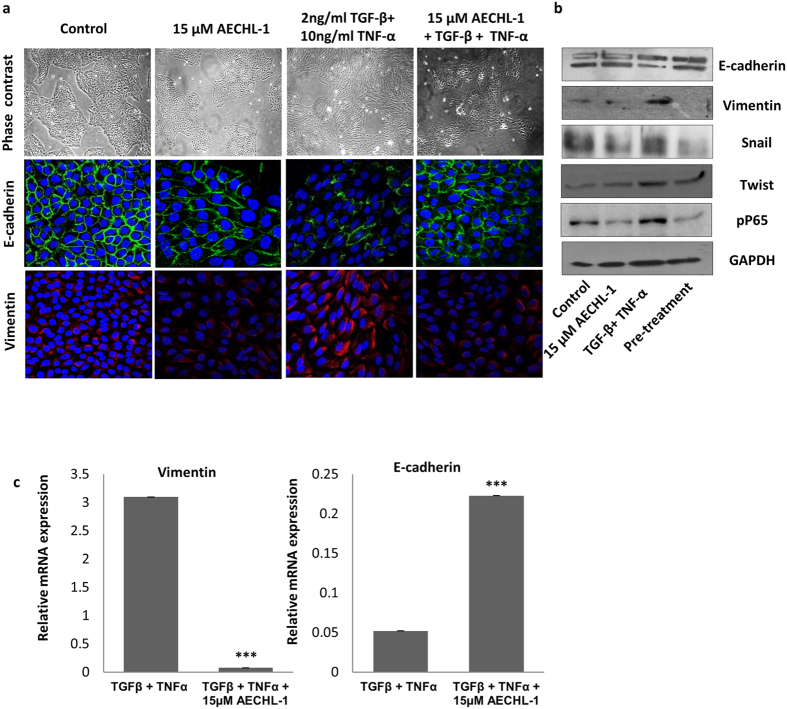
AECHL-1 prevents the acquisition of EMT phenotype in stimulated MCF 10 A cells. 10 ng/ml TNF-α and 2ng/ml TGF-β were used in EMT induction over a period of 3–7 days. Pre-treatment by AECHL-1 was for 18 h. (**a**) 3 days post induction, epithelial and mesenchymal markers were visualized by Immunofluorescence analysis using a confocal microscope (X 63). (**b**) Protein levels of E-cadherin, vimentin, Snail, Twist and pp65 were determined by Western blotting. The full length blots and densitometry analyses are given as supplementary Figure 1 and 2 respectively. Blots were stripped and reprobed for GAPDH to indicate equal loading. (**c**) Vimentin and E-cadherin levels were detected by total mRNA levels via qRT PCR. Images are representative of three independent experiments.

The remarkable ability demonstrated by AECHL-1 in cumulatively modulating various factors, responsible for observable changes in morphological and invasive properties, as well as metastasis inhibition led us to wonder whether AECHL-1 could prevent the onset of EMT, which follows similar tenets. Thus, EMT was induced in immortalized breast epithelial cell line MCF-10A by 2 ng/ml TGF-β and 10ng/ml TNF-α, over a period of 3–7 days. The cells were treated with 15 μM AECHL-1, 18 h prior to induction, and analyzed for epithelial and mesenchymal markers via Western blotting and Immunofluorescence assays. For a 7 day culture, media containing the appropriate quantities of the chemical EMT inducers was changed every 3 days. Acquisition of EMT phenotype was determined by the observation that these cells attained spindle shaped morphology losing their typical hexagonal shape (Fig. 5a).

Evidence of EMT was also confirmed by studying the status of E-cadherin, an epithelial marker and vimentin, a mesenchymal marker. Western blotting and confocal analysis revealed a decrease in E-cadherin accompanied by a concomitant increase in vimentin expression (Fig. 5a,b).

Pre-treatment with AECHL-1 prior to EMT induction prevented the acquisition of a mesenchymal phenotype by the MCF-10A cells. NF-κB is also known to mediate TNF-α induced EMT through upregulation of various transcriptional repressors such as ZEB1/2, TWIST and Snail (SNAI)^27^. AECHL-1 could also downregulate the expression of NF-κB regulatory subunit p65, Snail and Twist (Fig. 5b). Our qRT-PCR revealed an increase in Vimentin along with a decrease in E-cadherin in stimulated MCF-10A cells. AECHL-1 treatment could decrease the transcript levels of Vimentin and partially restore E-cadherin levels (Fig. 5c).

### AECHL-1 suppresses the cancer stem cell population *in vitro*

**Figure 6:**
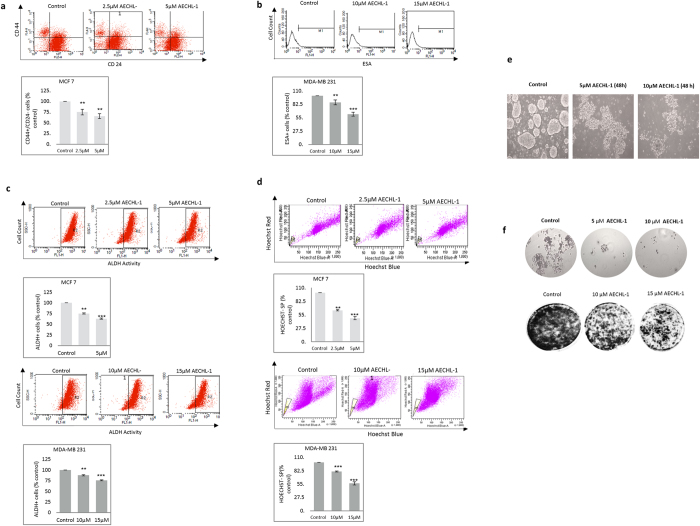
AECHL-1 suppresses breast cancer stem cell population *in vitro*. (**a**) AECHL-1 decreased the CD44^+^/CD24^−^ population in treated MCF 7 cells as detected by flow cytometry. (**b**) AECHL-1 reduced the ESA^+^ sub-population in MDA-MB-231 cells post treatment at 10 and 15 μM concentration of AECHL-1 as seen by flow cytometry. (**c**) The ALDH reducing population of cells was significantly suppressed by AECHL-1 as seen in MCF 7 and MDA-MB-231 via flow cytometry. (**d**) AECHL-1 inhibited the HOECHST 33342^−^ side population in MCF 7 and MDA-MB-231 cells. (**e**) Phase contrast images showing the inhibition of mammosphere formation in AECHL-1 treated MCF 7 cells 48 hours post treatment. AECHL-1 could significantly reduce the colony formation as seen in (**f**) MCF 7 cells and (**g**) MDA MB 231 cells 24 hours post-treatment as seen by phase contrast images of colonies stained with crystal violet. Columns, mean from three independent experiments; bars, SE. *P < 0.05; ***P < 0.001; versus control.

The process of EMT is closely associated with promoting the incidence of cancer stem cells in a tumor niche^28^ and as AECHL-1 could discourage EMT in breast cancer cells, we investigated its effect on the subpopulation of breast cancers cells exhibiting CSC like characteristics. Breast CSCs show a characteristic CD44^+^/CD24^−^/ESA^+^ phenotype, enhanced ALDH activity and possess a capability to form mammospheres in non-adherent cultures^29^. Also, ABC transporters enable these cells to efflux the chemotherapeutics out, providing them a distinct signature in the Hoechst Side Population Assay^30^. Thus, to analyze these parameters, cells were treated with AECHL-1 and 24 h post treatment, these trypsinized cells were subjected to different standardized assays. Immunophenotyping with CD44, CD24 and ESA yielded a significant decrease in the CD44^+^/CD24^−^ antigen positive cells in MCF7 (Fig. 6a) and ESA^+^ cells in MDA-MB-231 cells (Fig. 6b). A reduced ALDH activity was also noted in both these cell lines (Fig. 6c). AECHL-1 decreased the Hoechst 33342 effluxing side-population (SP) in MCF7 and MDA-MB-231 cells (Fig. 6d).

It was also observed that AECHL-1 treatment diminished the mammosphere forming capability in MCF7 cells, 48 h post-treatment (Fig. 6e). The colony formation propensity, which is directly correlated to the regenerative characteristic, was seen to be directly affected by AECHL-1 in MCF7 and MDA-MB-231 cells (Fig. 6f,g).

### AECHL-1 suppresses the cancer stem cell population *in vivo*

**Figure 7:**
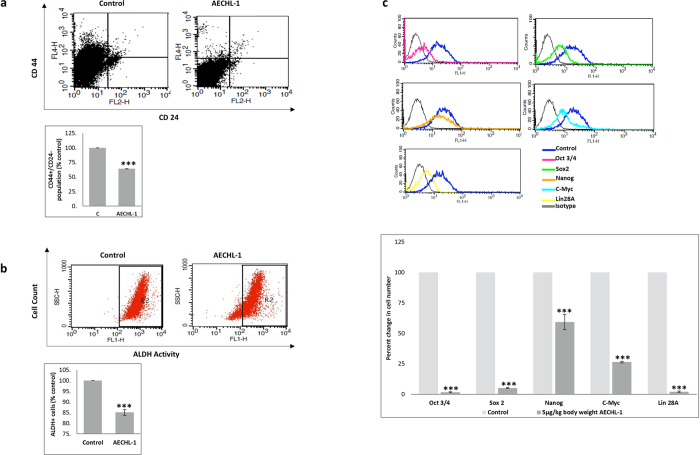
AECHL-1 suppresses breast cancer stem cell population *in vivo*. Cells isolated from xenograft tumors were live stained and subjected to flow cytometry which revealed AECHL-1 reduced the (**a**) CD44^+^/CD24^−^ population and (**b**) ALDH reducing population as well as brought about the decrease in (**c**) cancer stem cell markers oct3/4, sox2, nano, c-myc and lin28A.Columns, mean from three independent experiments; bars, SE. *P < 0.05; ***P < 0.001; versus control.

Xenograft tumors, obtained by injecting MCF7 cells in SCID mice, were lysed fresh after harvesting and live cells were analyzed for cancer stem cell population. CD44^+^/CD24^−^ Immunophenotyping revealed a significant decrease in their expression levels in tumors harvested from AECHL-1 treated mice as opposed to the tumors belonging to control mice (Fig. 7a). The ALDH reducing population also decreased significantly in AECHL-1 treated mice as compared to the control, as seen by Flow Cytometry (Fig. 7b).

Transcriptional regulators known for initiating and preserving cancer stem cell characteristics, viz. Oct3/4, Sox2, Nanog, C-myc and Lin28A^31^, were also seen to be drastically downregulated in AECHL-1 treated mice as compared to control, as observed by flow cytometry (Fig. 7c).

## Discussion

Metastasis requires clusters of tumor cells to make their way through the protein dense ECM and subsequently invade extra-tumoral tissues, including the vascular and lymphatic systems. These cancer cells efficiently exploit their altered signaling profiles to initiate the process of collective cell migration and invasion, which requires extensive cytoskeleton remodeling. Thus, therapeutic disruption of this process can derail the mechanisms integral to metastasis and provide an option for containing the devastating spread of cancer. In this study, our compound of interest, AECHL-1 was tested for its ability to interfere with this transformative process that tumor cells undergo in order to form metastatic lesions.

The pro-inflammatory cytokine TNF-α plays a significant role in driving the progression of tumorigenesis, influencing metastasis and regulating angiogenesis through the activation of multiple inter connected networks, including the upregulation of proteolytic enzymes such as MMP-9 and uPA via the NF-ĸB pathway^18^,^20^,^32^. However, despite TNF-α stimulation, we observed a marked reduction in the migratory and invasive potential of AECHL-1 treated MDA-MB-231 cells. This observation could be explained by the AECHL-1 mediated inhibition of nuclear translocation and down regulation of the active subunit of NF-ĸB, which is phosphorylated p65^19^,^27^, consequently effectuating a decrease in MMP-9 activity. TNF-α also has the ability to affect the cytoskeletal apparatus by associating with the Rho family of GTPases, including Cdc42 and Rac1^33^. Our studies indicated that the Rho family of cytoskeleton effector proteins could be a possible target of AECHL-1, since the TNF-α induced pro-migratory phenotype was abrogated by AECHL-1 treatment. A decrease in actin polymerization, reduction in active leading edge protrusions and perturbed localization of IQGAP-1 and WAVE-2 was observed on AECHL-1 treatment, with or without TNF-α induction. These changes in the expression and patterning of proteins related to cytoskeletal organization and assembly (ARP-2 and 3) could be an outcome of AECHL-1 interfering with the aberrantly over-stimulated signaling pathways, present in aggressive cancer cells. Thus, AECHL-1 could broaden its reach and disturb myriad functions ranging from cell survival to motility. Other molecules that have been implicated in TNF-α stimulated modulation of the cytoskeleton through the GTPases, include MAP kinases^34^,^35^, which were also found to be down regulated by AECHL-1 treatment, thus confirming our hypothesis that the principle proteins involved in cytoskeletal remodeling are actively targeted by AECHL-1. However, contrary to these results, Profilin, an actin binding protein, which increases actin filament turnover rates, was up regulated by AECHL-1 treatment in actively migrating cells. Further investigation into the reported literature revealed that in MDA-MB-231 cells, Profilin is usually present in insignificant amounts and acts as a tumor suppressor through the PTEN pathway^36^. Interestingly, in triple negative breast cancer patients, lower Profilin levels are synonymous with poor prognosis^37^.

The proteins involved in regulating the cytoskeletal apparatus also work in tandem with junctional proteins such as β-catenin and E-cadherin. Their dynamics influence junctional integrity affecting cell migration, invasion and polarity. β-catenin, the effector protein of the Wnt pathway, is responsible for maintaining cell-cell adhesive contact through α-actinin involvement, which connects it to the cytoskeleton^38^. On the other hand, its translocation to the nucleus, elicits a migratory response in these cells, setting in motion the acquisition of a more invasive phenotype along with a loss of a apico-basal polarity^39^. Also nuclear β-catenin is a co-activator to the Tcf/lef transcription factors which bind to the enhancer elements of genes falling under the Wnt pathway, including pro-invasive markers such as CD-44 and other pro-survival genes^26^. On AECHL-1 treatment, we observed a decrease in the nuclear localization and expression of β-catenin along with a down regulation of the phosphorylated form of its negative regulator GSK-3β, and maintenance of the activated GSK-3β at levels similar to that of the control cells. Further, an AECHL-1 mediated decrease in the secretion of TNF-α induced pro-angiogenic cytokines VEGF and bFGF contributed towards making the extracellular microenvironment less conducive for initiation of cell migration and invasion, thus complementing the inhibitory modalities effectuated by AECHL-1 on these mechanisms at the intracellular level.

Changes in cell morphology, polarity and invasiveness are indisputably the most important factors linked with the events of EMT. Our findings with AECHL-1 in this direction revealed a suppression of mesenchymal marker Vimentin along with a restoration of epithelial marker E-cadherin to functional levels, in MCF-10A breast epithelial cells, which had been induced to undergo EMT by TGF-β/TNF-α treatment. The transcription factors responsible for the induction and maintenance of EMT, namely, NF-κB, Snail and Twist were also found to be downregulated by AECHL-1, only in cells subjected to EMT. Also, evidence derived from these experiments indicate that AECHL-1 did not change the expression pattern of either group of markers or transcription factors responsible for EMT, in non-induced MCF-10A cells, hence strengthening our hypothesis that AECHL-1 did not affect cells with normal functioning or non-erratic signaling. Since TNF-α, through NF-κB, is known to regulate the expression and activity of β-catenin, Snail and GSK-3β^40^, our results indicate that NF-κB plays an indispensable role in implementing the anti-cancer program triggered by AECHL-1. A previous study concerning the apoptosis inducing mechanism of AECHL-1 too highlighted the involvement of NF-κB^16^.

A sub-population of cells within the tumor, known to be resistant to chemo/radiation therapy, contributes heavily towards tumor relapse, by facilitating metastatic growth and establishing secondary lesions. Their presence always resonates with poor prognosis in patients^41^. These cells have stem-cell like characteristics and are known to have distinct cellular and molecular signature which enables them to escape chemo/radiotherapy and attain the property of self-renewal^42^. Recently, these CSCs are shown to be intimately linked with the process of EMT, and studies have shown a direct correlation between EMT induction and a surge in the population of CSCs^42^,^43^. AECHL-1 treatment showed a significant decrease in the CSC sub-population *in vitro* as well as *in vivo*, thus strengthening our hypothesis that AECHL-1 could bring about an overall suppression of the disease.

In mice orthotropically injected with MDA-MB-231, AECHL-1 could decrease the tumor volume and down regulate all molecular players involved in invoking the invasive cytoskeletal phenotype. Surprisingly, Profilin in this case was found to be down regulated too, which could be attributed to a global protein down regulation observed in these tumors after one month of treatment, especially when they were already undergoing recession^16^. These, AECHL-1 mediated, inhibitory effects were also responsible for the decrease in presence of metastatic lesions in the lungs of animals, injected with MDA-MB-231 through the tail vein.

**Figure 8:**
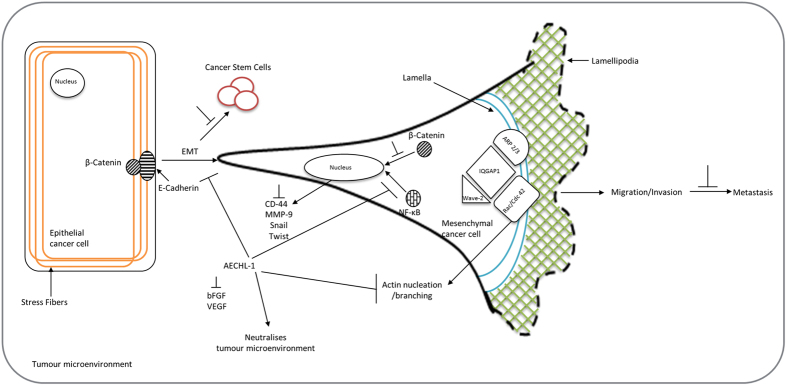
Schematic representation of possible mode of action shown by AECHL-1. AECHL-1 targets the nexus of migration-invasion-metastasis by disrupting the cytoskeletal dynamics and down regulating the actin-nucleation proteins. It also attacks the EMT machinery and associated CSC characteristics.

Overall, AECHL-1 launched a multi-targeted attack on metastatic breast cancer cells *in vitro* and *in vivo*, which closely resembles its activities in stimulated endothelial cells (Fig. 8). This diversified approach executed by AECHL-1 should be actively harnessed for development of anti-cancer leads, which in combination with appropriate regiments or alone would affect tumor development and disease progression in a comprehensive manner.

## Materials and Methods

### Reagents

Liebovitz’s L15 Medium and 0.5% Trypsin Phosphate Versene Glucose (TPVG) was purchased from HiMedia (USA). M171 Medium and Mammary Epithelial Growth Supplement (MEGS) were obtained from Life Technologies (USA). Fetal Bovine Serum (FBS), Penicillin, and Streptomycin (P&S) were purchased from Gibco (Grand island, NY, USA). All reagents were procured from Sigma-Aldrich (St. Louis, MO, USA) except for if otherwise mentioned. Primary antibodies for Immunofluorescence and Western Blotting were purchased from Cell Signaling Technologies (CST), USA and Santa Cruz Biotechnology (Santa Cruz, CA, USA). AlexaFluor secondary antibodies for Immunofluorescence and Flow Cytometry were obtained from Invitrogen (Life Technologies, USA). HRP-Linked anti-rabbit or anti-mouse secondary antibodies for Western Blotting were obtained from BioRad. All ELISA kits were purchased from R&D Systems, USA.

### Cell Culture

MDA-MB-231 and MCF7 cells were obtained from American Type Culture Collection (ATCC) and maintained in Leibovitz’s L15 Medium and DMEM respectively, supplemented with 10% Fetal Bovine Serum, FBS. MCF-10A cells, obtained from ATCC, were grown in M171 Medium supplemented with MEGS. Cultures were maintained at 37 °C with 5% CO_2_ in a humidified incubator.

### Wound healing assay

MDA-MB-231 cells were seeded in 24-well plates and grown up to nearly 100% confluency. The cells were scratched with a pipette tip to create wounds. Treatment with TNF-α (20 ng/ml) and AECHL-1 (15 μM), alone and in combination with each other was given in serum-free medium after scratch was made. Randomly chosen fields were photographed at 10X magnification with an inverted microscope, and the images were taken at identical locations at the indicated time points. Percent cell migration was calculated by comparing final gap width to initial gap width using image pro-plus. MDA-MB-231 cell migration from TNF-α treated wells at the final time point was normalized to 100% migration.

### Matrigel Invasion assay

MDA-MB-231 cells were suspended in serum-free culture medium and loaded onto Matrigel-coated inserts (BD Biosciences, USA), placed in a 24-well plate. The lower chamber, thus created, was filled with 500 μl 20% FCS (chemo-attractant) containing culture medium. After 18 h, the upper surface of the insert was swabbed with a cotton bud and invasive cells on the lower surface were fixed in 3.7% PFA. The inserts were then stained using 1% crystal violet and imaged (10X) using an inverted microscope (Nikon). Image J was used for counting the invasive cells.

### Detection of protein levels by Flow cytometry

Expression of αvβ3, CD24 and CD44 after AECHL-1 treatment were studied using flow cytometry. Cancer stem cell markers oct3/4, sox2, nanog, c-myc and lin28A were detected in xenograft tumor cells via flow cytometry as well. Cells from monolayers were trypsinized whereas tissue was subjected to dissociation by collagenase-III (1 mg/ml) treatment to obtain a single cell suspension, subsequently these cells were fixed with 3.7% PFA for 10 min on ice and incubated with the appropriate primary antibody (1:150) in 1% blocking buffer at 37 °C for 30 min followed by washing and binding with appropriate fluorescence tagged secondary antibody (AlexaFluor 488; 1:300) in 1% blocking buffer at 37 °C for 30 min. Cells analyzed for expression of surface marker analysis were not subjected to PFA fixing and were stained live. Cells were then washed twice with PBS and immediately acquired using FACSCalibur. Appropriate isotype controls were used to subtract background fluorescence intensities. Analysis was carried out using Cell Quest Pro software.

### Nuclear Cytoplasmic Extraction

Cells were trypsinized using 0.5% TPVG (HiMedia) and incubated in Hypotonic buffer [10 mM HEPES pH 7.9, 10 mM KCl, 2 mM MgCl2, 0.1 mM EGTA, 0.2 mM PMSF, protease inhibitor cocktail (Roche), sodium pyrophosphate (0.2 mM), fluoride (10 mM) and orthovanadate (50 mM)] for 15 min on ice. The cytosolic fraction was collected by centrifugation at 5000 g for 15 min at 4 °C. The pellet was washed once in hypotonic buffer and resuspended in cell lysis buffer [50 mM Tris pH 7.4, 5 mM EDTA, 250 mM sodium chloride, 50 mM sodium fluoride, 0.5 mM sodium vandate and 0.5% w/v Triton X-100]. The resuspended pellet was incubated for 30 min at 4 °C with rotation shaking. Nuclear extracts were centrifuged at 13000 g for 15 min at 4 °C.

### Western Blot Analysis

Whole cell protein lysates were prepared using RIPA buffer containing 0.2% SDS, 1 mM EDTA, 1% NP-40, 0.5% sodium deoxycholate, 1 mM sodium orthovanadate, 1 mM sodium fluoride, 1 mM PMSF and EDTA-free mini-complete protease inhibitor cocktail tablet (Roche). Tissue samples or cells were homogenized in RIPA Lysis Buffer. The protein concentration was determined using Bradford’s Reagent (BioRad). 40 µg of protein was subjected to polyacrylamide gel electrophoresis and transferred on to a nitrocellulose membrane (Merck Millipore). Membranes were incubated with respective primary antibody dilutions prepared in Tris Buffered Saline (TBS)-Tween for 2 h at room temperature or overnight at 4 °C. Membranes were then washed in TBS-Tween and incubated with secondary antibodies anti-rabbit IgG–HRP (BioRad) or anti mouse IgG-HRP (BioRad) diluted (1:10.000) in TBS-Tween for 1 h at room temperature. Protein-antibody complexes were detected by Substrate Detection Kit (Thermofischer, USA). Quantification of signal was done via densitometry analysis, using the Image J software. Actin was used as loading control for whole cell lysates and Lamin A/C and GAPDH were used as loading controls for nuclear and cytoplasmic fractions respectively.

### RNA Extraction, qRT-PCR

Total RNA was isolated from cells and tissue samples using TRIzol reagent (Sigma-Aldrich, USA) and reverse transcribed using Thermoscript cDNA synthesis kit (Life Technologies) as per manufacturer’s protocol. cDNA was quantified by a spectrophotometer using NanoDrop Analysis. Approximately 600ng of cDNA was subjected to qRT PCR using SYBR green (Life Technologies) according to manufacturer’s instructions on an Applied Biosystems real-time thermocycler. The profile of thermal cycling consisted of initial denaturation at 95 °C for 2 min, and 40 cycles at 95 °C for 15 s and 60 °C for 45 s for primer annealing and extension. Melting curve analysis was used to determine the specific PCR products. All primers used for Real-Time PCR analyses were synthesized by Eurofins Genomics India Pvt. Actin was used as a positive control and appropriate negative control was used to validate the reaction. The list of primers is given below as Table 1. The changes in the threshold cycle (CT) values were calculated by the equation ΔCT = CT (target)  − CT (endogenous control) and fold difference was calculated as 2^−Δ (ΔCT)^.

### Immunofluorescence Assay and Confocal Imaging

Cells were fixed using 3.7% PFA followed by permeabilization (for intracellular proteins only) with 0.2% Triton X-100. 5% BSA was used for blocking followed by staining with protein specific primary antibody and Alexa Fluor secondary antibody (Invitrogen). DAPI was used for nuclear staining. Cells were visualized on a Zeiss LSM510 META (Carl Zeiss, Germany) and Leica SP5 II system (Leica microsystems, Germany) and images were analyzed using LSM 5 Image Browser software.

### Gelatin Zymography

MDA-MB-231 cells were cultured to 70% confluence followed by treatment with AECHL-1 for 18 h. Cell free supernatants of culture were used to analyze MMP-9 levels using Gelatin Zymography. Aliquots of media supernatant were diluted 1:1 in zymography sample buffer [62.5 mM Tris–HCl (pH 6.8), 8.8% glycerol, 2% (w/v) SDS, 0.05% bromophenol blue] and electrophoresed on a 7.5% SDS-polyacrylamide gel containing 1% gelatin, at 4° C. After Electrophoresis, the gel was washed twice for 30 min with 2.5% Triton X-100, to remove SDS followed by incubating in renaturation Buffer (1 M Tris HCl, pH 7.6, NaCl, CaCl_2_ and NaN_3_) for 18 h at 37 °C. Gels were stained with Coomassie Brillant Blue (0.1%w/v) and destained in 30% methanol, 10% acetic acid. Gels were visualized in Gel Documentation System (BioRad) and images were analyzed using GeneSnap (BioRad) and Image J Software.

### ELISA

ELISA kits (R&D Systems) were used to detect human Basic fibroblast growth factor (bFGF) and Vascular Endothelial Growth Factor (VEGF). MDA-MB-231 cells were treated with AECHL-1 for 18 h and cell free supernatants were used to detect the levels of bFGF and VEGF by following the manufacturer’s protocol.

### Animal Experiments Ethical Statement

All experiments were performed in accordance with relevant guidelines and regulations as per the Institutional Animal Ethics Committee (IAEC) under reference number EAF/2013/B-217 dated 29/03/2010 of National Centre for Cell Science (NCCS). All experimental protocols were approved by IAEC, a committee constituted at NCCS, as per regulations of Committee for the Purpose of Control and Supervision of Experiments and Animals (CPCSEA), Government of India.

### Tail Vein Metastasis

Metastasis was assessed by injecting 1 × 10^6^ MDA-MB-231 cells suspended in 150 μL sterile Phosphate Buffer Saline (PBS), in the tail vein of 6–8 week old female SCID mice. Animals were treated intra-peritoneally (i.p.) with PBS or AECHL-1 5 μg/kg body weight of the animal for 10 days following tail vein injection. Metastasis to the lung was detected by generating cryosections of lungs, after 4 weeks. Tissue samples were fixed in OCT Compound at −20 °C overnight. The fixed tissue samples were then sliced into sections not larger than 8 μm using a Shandon microtome. Metastatic foci were identified by H&E staining of the lung sections. Quantification was carried out by measuring the metastatic index in three random fields per three lung sections per animal (n = 5). Metastatic index was determined by calculating the ratio of “area covered by metastatic foci per section” to “total section area”.

### MCF7 and MDA-MB-231 xenograft studies

Six-week-old female SCID mice were injected subcutaneously into the dorsolateral flank with 2 × 10^6^ MCF7 or MDA-MB-231 cells. When tumor volume reached visible proportions, animals were treated intra-peritoneal (i.p.) with PBS or AECHL-1,5 μg/kg body weight of the animal for 10 days. After harvesting tumors, part of the tissue was stored at −80 °C for flow cytometry or subjected to Western blotting.

### HOECHST 33342 Side Population Analysis

Cells were plated in 60 mm petridish as 2.5 × 10^5^ cells/petridish and were treated with increasing concentrations of AECHL-1 at 70% confluence for 24 h. Cells were then trypsinized and stained with HOECHST 33342 for 90 min at 37 °C. The cells were then analyzed using FACS Aria UV laser within 30 min of staining. Verapamil was used as an inhibitor control to back-gate the side population cells. Analysis was carried out using the FACS Diva software.

### ALDH Assay

ALDH reducing population was analyzed using the ALDEFLUOR Kit by Stemcell Technologies, USA as per the manufacturer’s protocol. Analysis was carried out using the Cell Quest Pro software.

### Statistical Analysis

Significant differences were analyzed using the Student t test and two-tailed distribution. Results were considered to be statistically significant if P < 0.05 and were expressed as mean ± SE between triplicate experiments performed thrice. All statistical comparisons were made relative to untreated controls and significance of differences is indicated as *P < 0.05 and **P < 0.01.

## Additional Information

**How to cite this article**: Dasgupta, A. *et al*. AECHL-1 targets breast cancer progression via inhibition of metastasis, prevention of EMT and suppression of Cancer Stem Cell characteristics. *Sci. Rep.* **6**, 38045; doi: 10.1038/srep38045 (2016).

**Publisher's note:** Springer Nature remains neutral with regard to jurisdictional claims in published maps and institutional affiliations.

## Supplementary Material

Supplementary Information

## Figures and Tables

**Table 1:** Primer Sequences.

E-cadherin-F	5′-CCTGGGACTCCACCTACAGA-3′
E-cadherin-R	5′-TGGATTCCAGAAACGGAGGC-3′
Vimentin-F	5′-TGCCCTTAAAGGAACCAATG-3′
Vimentin-R	5′-CTCAATGTCAAGGGCCATCT-3′
Actin-F	5′-AGCCTCTGATCTGTGCAGCG-3′
Actin-R	5′-TGACAGACCCGCAAGACAAA-3′
