# Supplementary material for: AECHL-1 targets breast cancer progression via inhibition of metastasis, prevention of EMT and suppression of Cancer Stem Cell characteristics
Source: Sci Rep. 2016 Dec 15;6:38045. doi: 10.1038/srep38045 (PMC5156909; doi:10.1038/srep38045)

AECHL-1 targets breast cancer progression via inhibition of metastasis, prevention of EMT and suppression of Cancer Stem Cell characteristics

Aparajita Dasgupta[1,+], Mithila A. Sawant[1,+], Gayatri Kavishwar[1,+], Manish Lavhale[2], Sandhya Sitasawad[1,*]

*Corresponding Author
Sandhya L. Sitasawad
Scientist-F
National Centre for Cell Science, NCCS Complex,
S.P. Pune University, Ganeshkhind, Pune 411007, Maharashtra, India
Phone: +91-20-25708148
Fax: +91-20- 25692259
Email: ssitaswad@nccs.res.in

Supplementary Information:

Supplementary Figure 1-Original Western Blots (Full Length)

Supplementary Figure 2-Densitometry Analyses of Western Blots

Supplementary Figure 1

Full Blots for Figure 2:

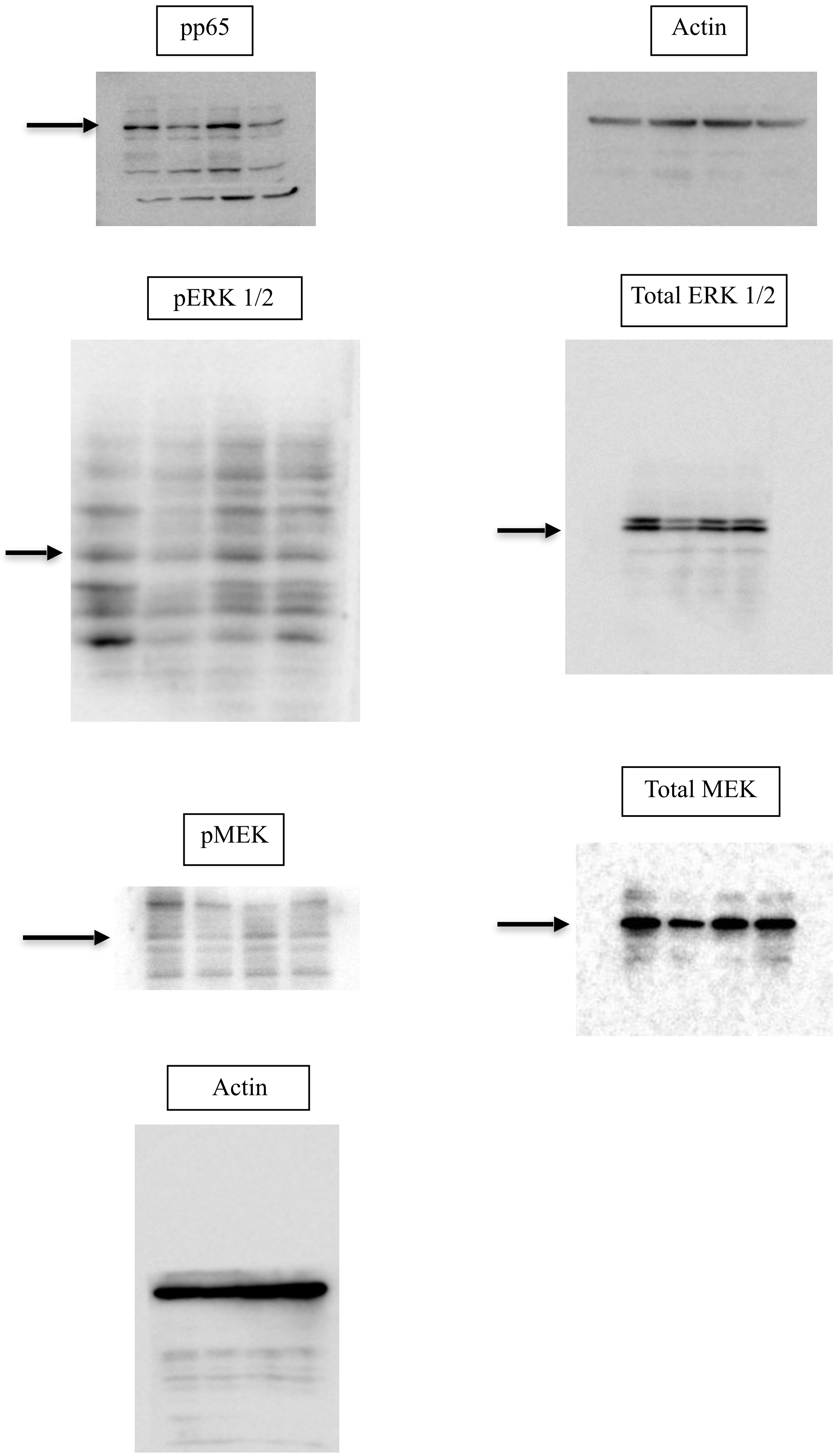

Supplementary Figure 1 (contd.)

Full Blots for Figure 3:

Profilin

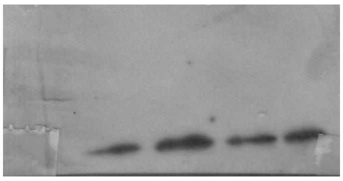

Wave2

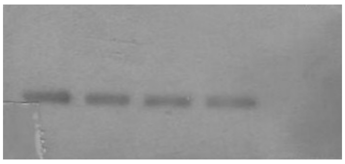

Arp2

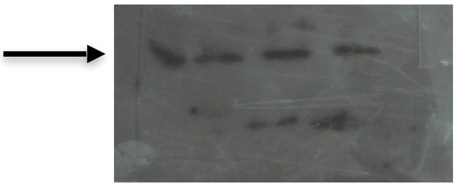

Arp3

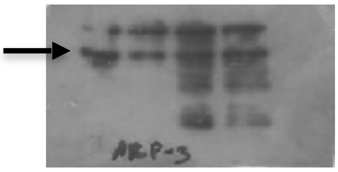

GAPDH

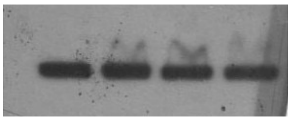

pRac/cdc42

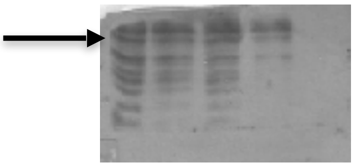

Total Rac/cdc42

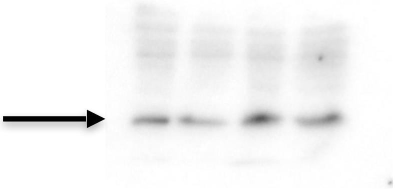

GAPDH

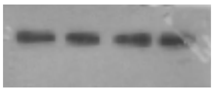

Supplementary Figure 1 (contd.)

Full Blots for Figure 3: (contd.)

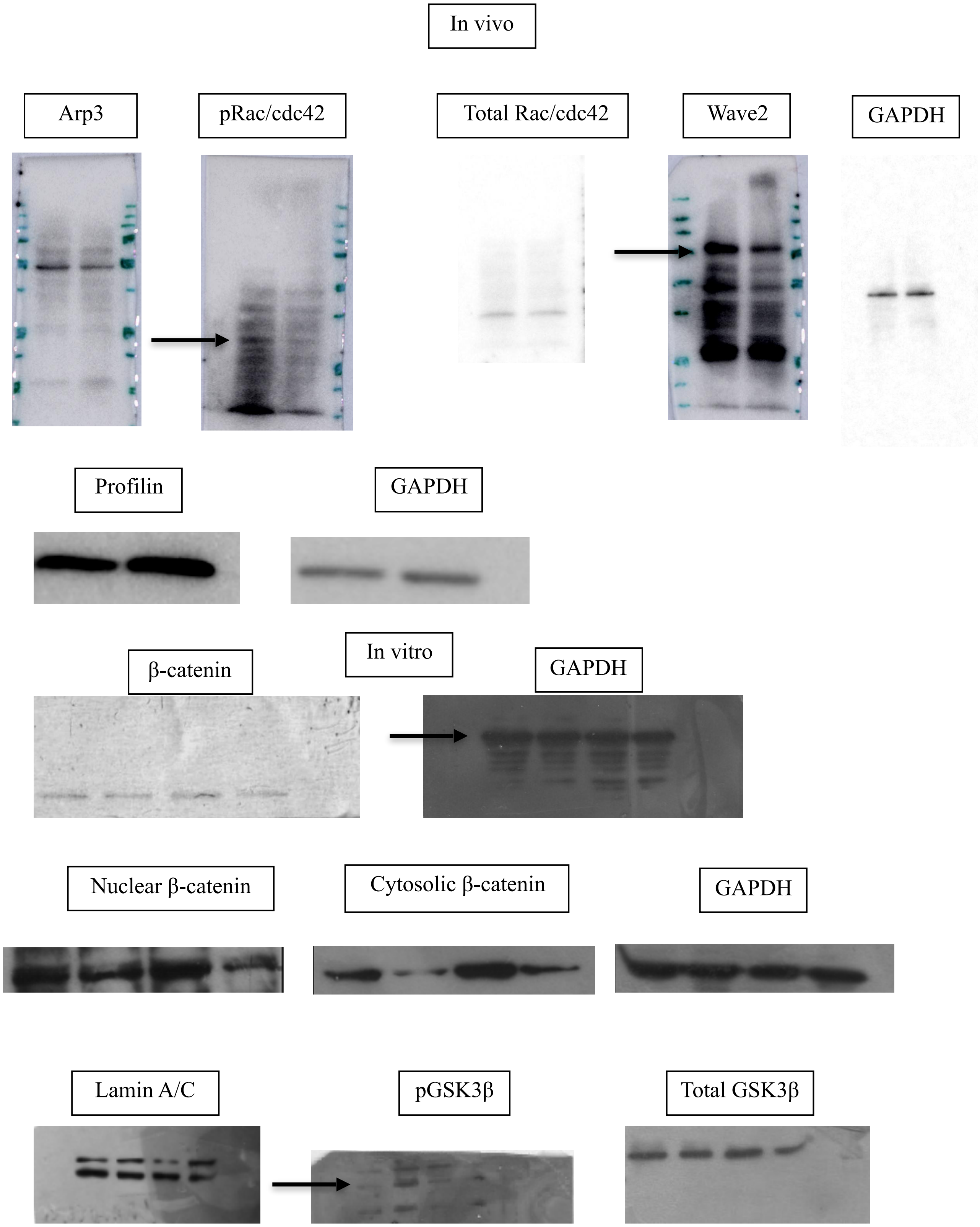

Supplementary Figure 1 (contd.)

Full Blots for Figure 5:

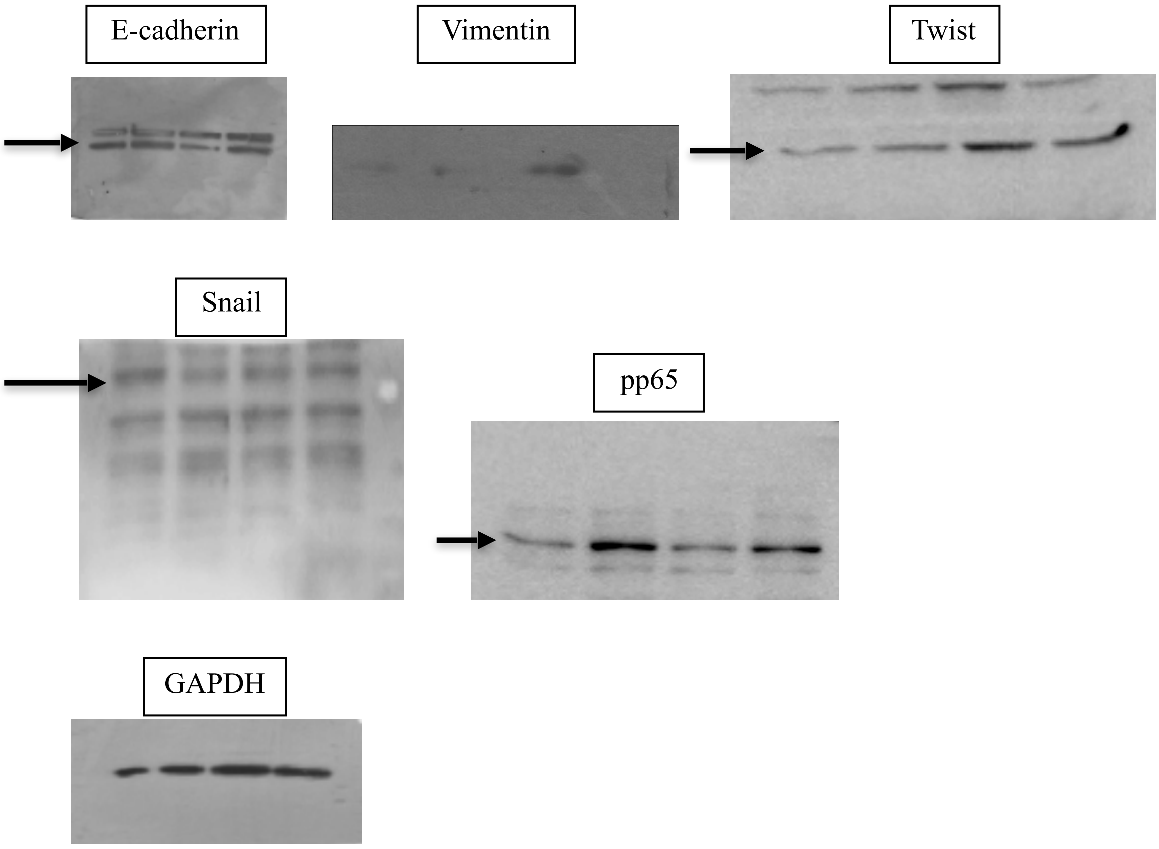

Supplementary Figure 2

Densitometry Analysis for Blots:

Figure 2

Figure 2a

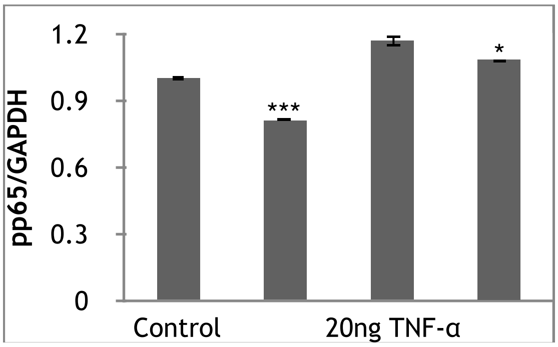

Figure 2c

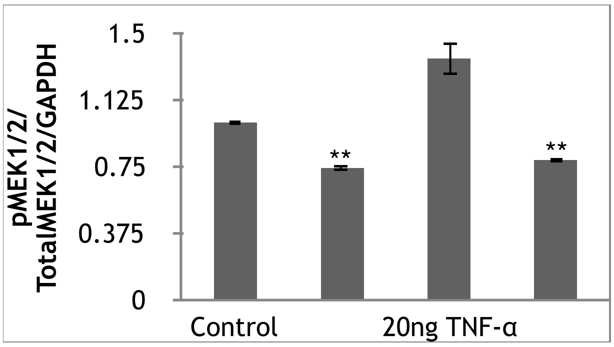

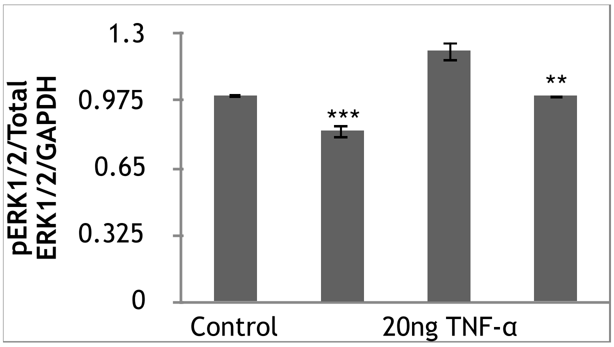

Supplementary Figure 2 (contd.)

Figure 3a

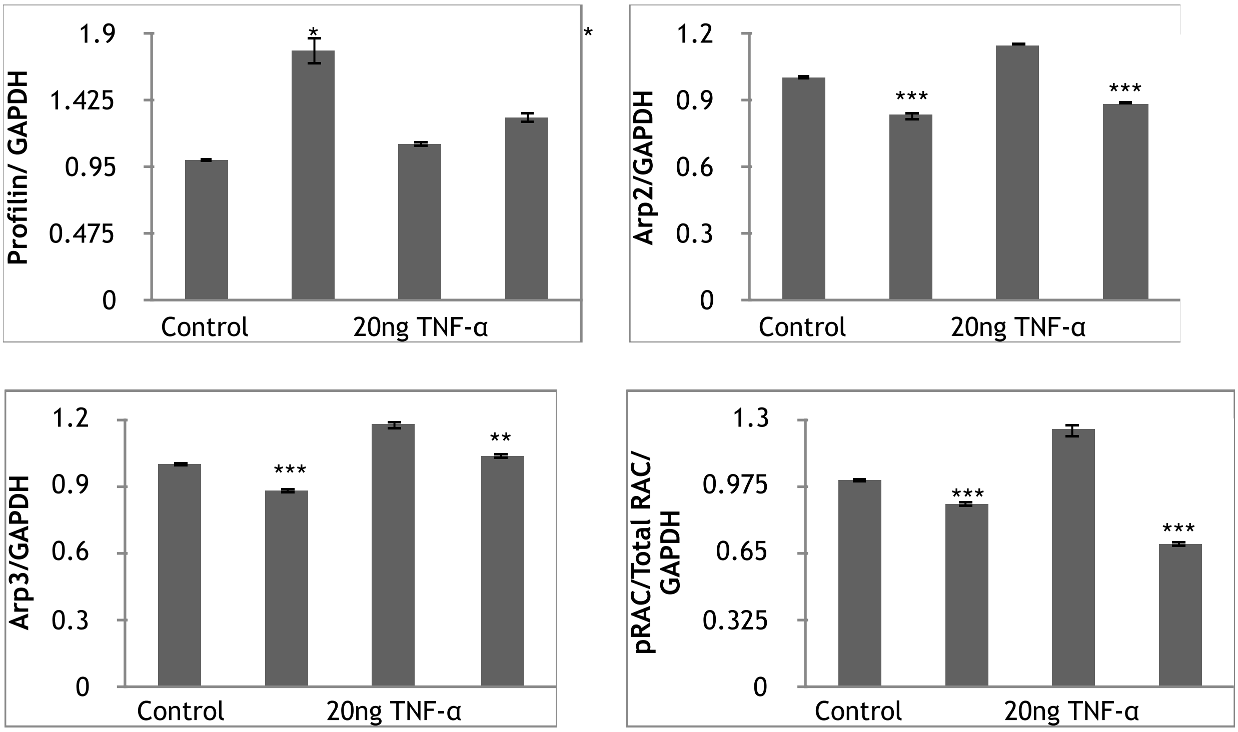

Figure 3e

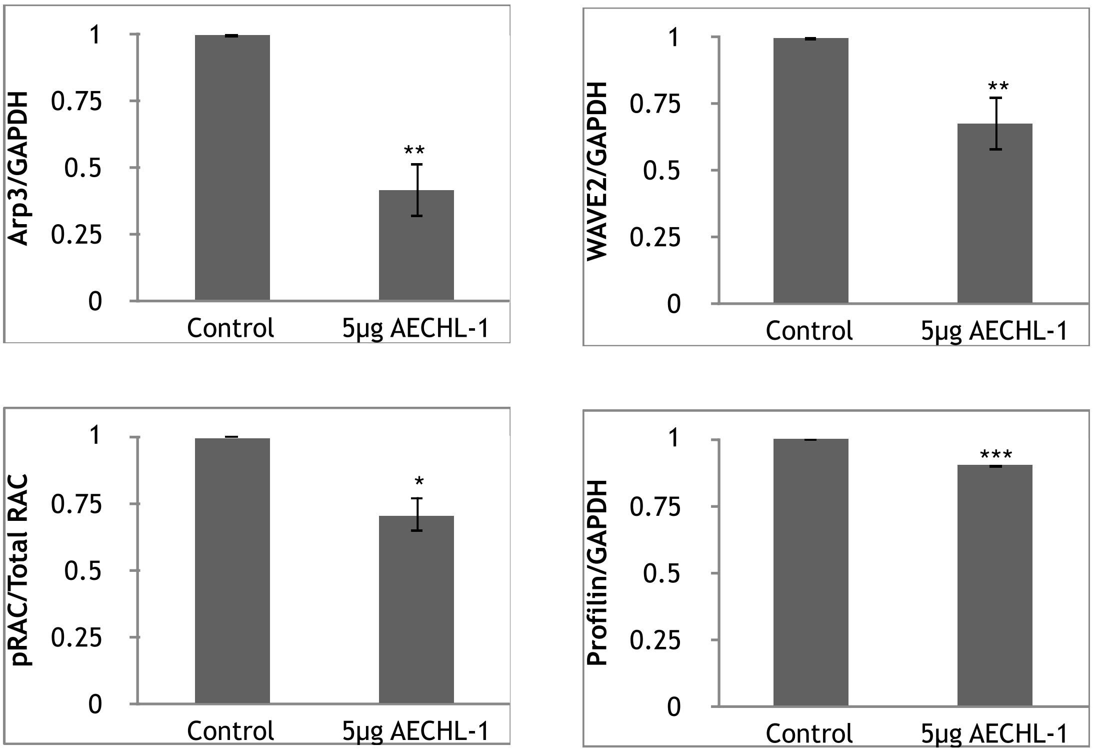

Supplementary Figure 2 (contd.)

Figure 3f

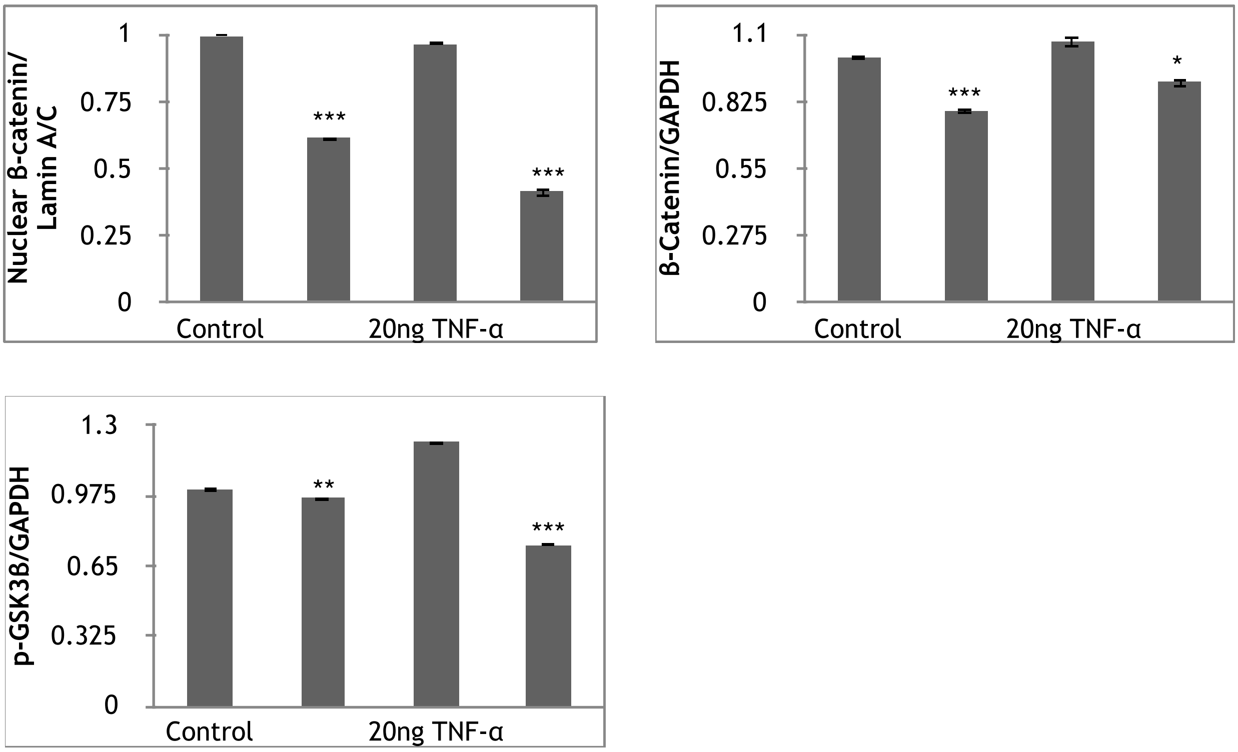

Supplementary Figure 2 (contd.)

Figure 5b

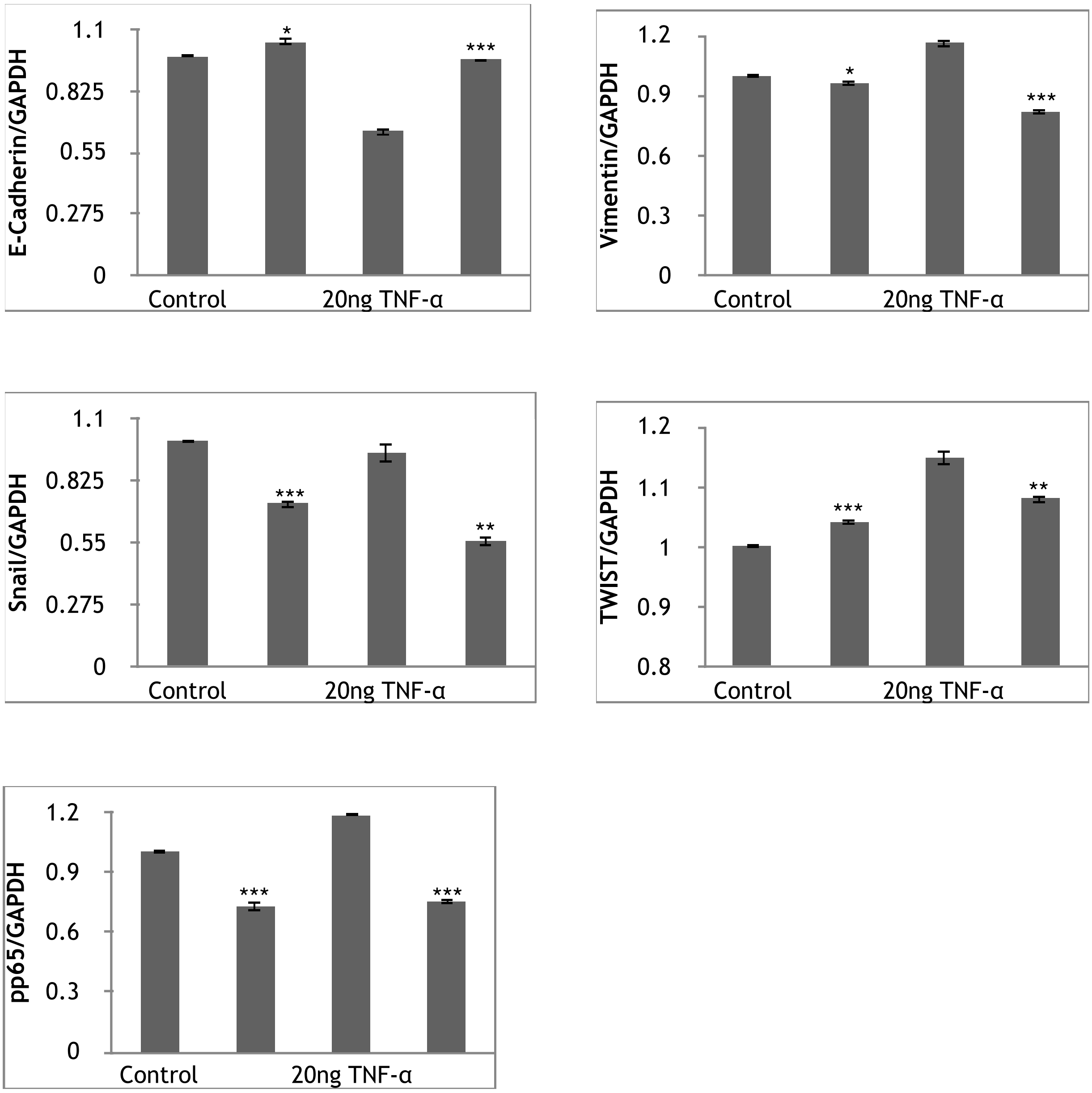

Supplement: Supplementary Information [file srep38045-s1.pdf]
